# CUL4B Upregulates RUNX2 to Promote the Osteogenic Differentiation of Human Periodontal Ligament Stem Cells by Epigenetically Repressing the Expression of miR-320c and miR-372/373-3p

**DOI:** 10.3389/fcell.2022.921663

**Published:** 2022-06-16

**Authors:** Jun Mi, Shuangshuang Wang, Panpan Liu, Chang Liu, Dexuan Zhuang, Xue Leng, Qun Zhang, Fuxiang Bai, Qiang Feng, Xunwei Wu

**Affiliations:** ^1^ Department of Tissue Engineering and Regeneration, School and Hospital of Stomatology, Cheeloo College of Medicine, Shandong University and Shandong Key Laboratory of Oral Tissue Regeneration and, Shandong Engineering Laboratory for Dental Materials and Oral Tissue Regeneration, Jinan, China; ^2^ Shenzhen Research Institute of Shandong University, Shenzhen, China; ^3^ Department of Pediatrics Dentistry, Department of Preventive Dentistry, School and Hospital of Stomatology, Cheeloo College of Medicine, Shandong University and Shandong Key Laboratory of Oral Tissue Regeneration and, Shandong Engineering Laboratory for Dental Materials and Oral Tissue Regeneration, Jinan, China; ^4^ Department of Human Microbiome, School and Hospital of Stomatology, Cheeloo College of Medicine, Shandong University and Shandong Key Laboratory of Oral Tissue Regeneration and, Shandong Engineering Laboratory for Dental Materials and Oral Tissue Regeneration, Jinan, China; ^5^ Engineering Laboratory for Biomaterials and Tissue Regeneration, Ningbo Stomatology Hospital, Savaid Stomatology School, Hangzhou Medical College, Ningbo, China; ^6^ Suzhou Research Institute, Shandong University, Suzhou, China

**Keywords:** periodontal ligament stem cells, osteogenic differentiation, CUL4B, miRNAs, RUNX2

## Abstract

Mesenchymal stem cells (MSCs) within the periodontal ligament (PDL), termed periodontal ligament stem cells (PDLSCs), have a self-renewing capability and a multidirectional differentiation potential. The molecular mechanisms that regulate multidirectional differentiation, such as the osteogenic differentiation of PDLSCs, remain to be elucidated. Cullin 4B (CUL4B), which assembles the CUL4B-RING ubiquitin ligase (CRL4B) complex, is involved in regulating a variety of developmental and physiological processes including the skeletal development and stemness of cancer stem cells. However, nothing is known about the possible role of CUL4B in the osteogenic differentiation of PDLSCs. Here, we found that knockdown of CUL4B decreased the proliferation, migration, stemness and osteogenic differentiation ability of PDLSCs. Mechanistically, we demonstrate that CUL4B cooperates with the PRC2 complex to repress the expression of miR-320c and miR-372/373-3p, which results in the upregulation of RUNX2, a master transcription factor (TF) that regulates osteogenic differentiation. In brief, the present study reveals the role of CUL4B as a new regulator of osteogenic differentiation in PDLSCs.

## 1 Introduction

MSCs have been widely used as basic biomaterials in tissue engineering due to their self-renewing ability and multidirectional differentiation potential. Periodontal ligament stem cells (PDLSCs), MSCs derived from the periodontal ligament, play an important role in dental tissue regeneration as well as tooth development, which show multiple differentiation and self-renewing capabilities (Seo et al., 2004; Iwasaki et al., 2013). PDLSC transplantation therapies have been reported to be involved in the regeneration of new bone, cementum/PDL-like structure, peripheral nerves and new cementum (Seo et al., 2004). Owing to the advantages of their availability, rapid culture expansion and hypoimmunogenicity (Feng et al., 2010), PDLSCs are not only widely used as favorable seed cells for periodontal tissues and bone regeneration but also as a cell model to study the self-renewing ability and multidirectional differentiation especially osteogenic differentiation (Liu et al., 2008; Mrozik et al., 2013).

Osteogenic differentiation is regulated by a diverse set of factors including hormones, TF and growth factors (Chen et al., 2012; Bruderer et al., 2014). RUNX2 is one of the earliest markers and is known as the master TF regulating osteogenic differentiation (Komori et al., 1997; Stein et al., 2004). RUNX2 is a TF that has the ability to upregulate ColeI, ALP and OPN genes (Fakhry et al., 2013). The regulation of RUNX2 is essential in osteogenic differentiation (Komori, 2010).

MiRNAs are small non-coding RNAs that are 17–25 nucleotides long. miRNAs regulate the expression of target genes at the post-transcriptional level by hybridizing with their target mRNAs at the 3′untranslated region (3′UTR), consequently silencing the target gene(s) and thereby controlling target protein biosynthesis (Wahid et al., 2010; Vimalraj and Selvamurugan, 2013). In osteoblasts, miRNAs play a pivotal role in the post-transcriptional regulation of genes that participate in differentiation. A number of miRNAs have been identified that regulate osteogenic differentiation via RUNX2 directly or indirectly (Narayanan et al., 2019).

CUL4B functions as a scaffold protein in the CUL4B-RING E3 ligase complex (CRL4B), which includes DDB1, RBX1 and substrate receptors (Jackson and Xiong, 2009; Hu et al., 2012; Zhao et al., 2015; Li et al., 2017). Previously, we showed that the constitutive knockdown of CUL4B inhibits the proliferation of a variety of cancer cells and contributes to cancer stemness in colorectal cancer and bladder cancer (Zou et al., 2009; Zou et al., 2013; Mi et al., 2017; Li et al., 2020; Liu et al., 2020). Mutations in human CUL4B cause intellectual disability (Tarpey et al., 2007; Zou et al., 2007), and patients with CUL4B mutations manifest disruptions in skeletal development such as short stature and brachydactyly (Badura-Stronka et al., 2010; Isidor et al., 2010; Kerzendorfer et al., 2011; Ravn et al., 2012). Bone formation is the basis of skeletal development, which implies that CUL4B could be involved in regulating the formation of mineralized bone. Osteogenesis is the process of new bone formation and there are distinct stages involved in the cells proliferate, grow and differentiate. Although accumulating evidence indicates that CUL4B exerts biological functions in various cancers, how it contributes to skeletal development and the biological properties of MSCs, as well as its role in osteogenesis, is still poorly defined. Given the advantages of PDLSCs mentioned above, in this study, we investigated the role of CUL4B in PDLSCs and demonstrate the effects of CUL4B on the proliferation, apoptosis, migration, stemness and osteogenic differentiation of PDLSCs.

## 2 Materials and Methods

### 2.1 Isolation, Culture and Manipulation of Periodontal Ligament Stem Cells

Human PDLSCs were isolated from healthy periodontal ligament (PDL) tissues of teeth extracted for orthodontic reasons. PDL tissues were cut into small pieces, which were detached for 45 min by 3 mg/L type I collagenase and 4 mg/ml dispase II suspended in α-modified Eagle’s medium (α-MEM, HyClone, South Logan, UT, United States) containing 10% fetal bovine serum (FBS, Gibco, Grand Island, NY, United States) and 1% penicillin-streptomycin antibiotic mixture (Invitrogen, Carlsbad, CA, United States). The cell suspensions were implanted into 10 cm tissue culture dishes and incubated at 37°C and 5% CO_2_. The non-adherent cell suspensions were removed 3 days later, and the basic medium was changed every 2–3 days. When MSC-like colonies had grown for 10–14 days, monoclones were selected by the limiting dilution method, and were then expanded for subsequent experiments.

Stable CUL4B-knockdown and -control cells were generated as described previously (Mi et al., 2017). Stable CUL4B-overexpression and -control cells were generated as described previously (Liu et al., 2020). miR-320c and miR-372/373-3p Sponge vectors were constructed by Abiotech (Abiotech, Jinan, China) ligating the miR-320c and miR-372/373-3p sponge fragments into the lentiviral vector pLent-EF1a-Puro-CMV-GFP-mirRNA sponge (Abiotech, pLV100016-KD). The pLVX-IRES-Puro-CUL4B (ΔNLS) was generated by subcloning the fragment from pCMV-Tag2B-CUL4B (ΔNLS). Lentiviral transductions were performed as previously described (Zou et al., 2013).

All experiments in this study were approved by the Medical Ethical Committee of School of Stomatology, Shandong University (No. GR201907). PDL tissue donors in the study signed an informed consent allowing the use of their PDL tissues for scientific research.

### 2.2 *In vitro* Proliferation Assays

Cell viability and proliferation were measured using Cell Counting Kit-8 (CCK-8) assays (Dojindo Laboratories, Kumamoto, Japan), colony formation assays and Cell-Light EdU DNA Cell Proliferation (EdU) assays (Ribobio, Guangzhou, China). For CCK-8 assays, cells were inoculated in 96-well plates, 10 μl CCK-8 was added to each well for 60 min, after which the absorption values were determined at 450 nm. The colony formation and EdU labelling assays were performed as previously described (Mi et al., 2017; Qi et al., 2019).

### 2.3 Wound-Healing and Transwell Migration Assays

To assess migration capacities of PDLSCs, wound-healing and transwell migration assays were performed as previously described (Mi et al., 2017).

### 2.4 Western Blot Analysis and Antibodies

Western blots were performed as described previously (Zou et al., 2013). Antibodies used in this study are listed in Supplementary Table S3.

### 2.5 Flow Cytometry (FACS)

The surface markers of PDLSCs were examined using a flow cytometer (Calibur, BD Biosciences). 1 × 10^6^ PDLSCs were incubated with following luorochrome-conjugated antibodies: anti-CD44-APC (Cat. MA1-10,226, Invitrogen), anti-CD105-PE (Cat. 12–1057–42, Invitrogen), anti-CD90-PE (Cat. 12–0909–42, Invitrogen), anti-CD45-FITC (Cat. 14–9457–95, Invitrogen), anti-CD19-APC (Cat. 17–0199–42, Invitrogen) and anti-CD14-PerCP (Cat. 450,149–42, Invitrogen) for 1 h at 4°C in the dark and were then analyzed after washing in PBS.

The percentage of cell apoptosis was determined by FACS. Apoptotic PDLSCs were detected according to the instructions of the AnnexinV-APC/7-AAD apoptosis detection kit (BestBio, Shanghan, China). Briefly, PDLSCs were trypsinized and the resuspended. PDLSCs were washed with PBS and stained with annexin V-APC and 7-AAD. The cells were then analyzed by flow cytometry (CytoFLEX; Beckman Coulter, Brea, CA, United States).

### 2.6 RNA Extraction, qRT-PCR and Chromatin Immunoprecipitation Assays

Extraction of total RNAs and qRT-PCR were performed as described previously (Zou et al., 2013). Primer sequences for qRT-PCR used in this study are listed in Supplementary Table S4. All primers used for miRNA qRT-PCR were purchased from GeneCopoeia, Guangzhou, China. Chromatin immunoprecipitation (ChIP) assays were performed as described previously (Hu et al., 2012). Primers and antibodies used in this study are listed in Supplementary Tables S5, S6.

### 2.7 ALP Activity Assay

ALP activity was measured according to the instructions of the ALP Activity Kit (Nanjing Jiancheng Bioengineering Institute, Nanjing, China). Briefly, cells were seeded in 6-well plates at a density of 2 × l0^5^ cells/well in osteogenic-inductive medium. After induction, cells were scraped into 1% Triton X-100, sonicated and centrifuged at 12,000 g for 10 min. The absorbance at 520 nm wavelength was measured using a microplate reader (SPECTROstar Nano; BMG Labtech).

### 2.8 Alizarin Red S Staining

Virus-infected PDLSCs (passages 3–5) were plated in 6-well plates at a density of 2 × l0^5^ cells/well and were cultured in osteogenic-inductive medium. After 3 weeks of induction, cells were rinsed with PBS and fixed with 70% ethanol, then stained with 1% Alizarin Red S (pH 4.2; Sigma-Aldrich) for 5 min. To measure the concentration of calcium, 10% (w/v) cetylpyridinium chloride (CPC; Sigma-Aldrich) and 10 mM sodium phosphate solution were added to the stained dishes for 10 min at room temperature, and then were quantified using a spectrometer at 562 nm wavelength.

### 2.9 Plasmids and Luciferase Assays

Wild-type and mutant RUNX2 3′- UTR vectors were constructed by Abiotech (Abiotech, Jinan, China). Dual luciferase assays were performed as previously described (Qi et al., 2019).

### 2.10 Spheroid Formation Assays

Single cells were plated in ultra-low 6-well attachment plates (Corning, Lowell, MA, United States) in H-DMEM/F12 medium supplemented with 1% penicillin-streptomycin antibiotic mixture, 1% (v/v) B27 (Gibco, Grand Island, NY, United States), 5 μg/ml insulin (Sigma-Aldrich), 20 ng/ml EGF (Gibco, Grand Island, NY, United States) and 20 ng/ml bFGF (Sigma-Aldrich). After 7–10 days, clonal spheres with a diameter over 50 μm were counted using an optical microscope.

### 2.11 Statistical Analysis

Statistical analyses were performed using an unpaired Student’s t test to calculate two-tailed *p* values between two groups. Differences were considered significant at *p* < 0.05. Data are reported as means ± SEM.

## 3 Results

### 3.1 Cullin 4B Promotes Proliferation and Migration of Periodontal Ligament Stem Cells

**FIGURE 1:**
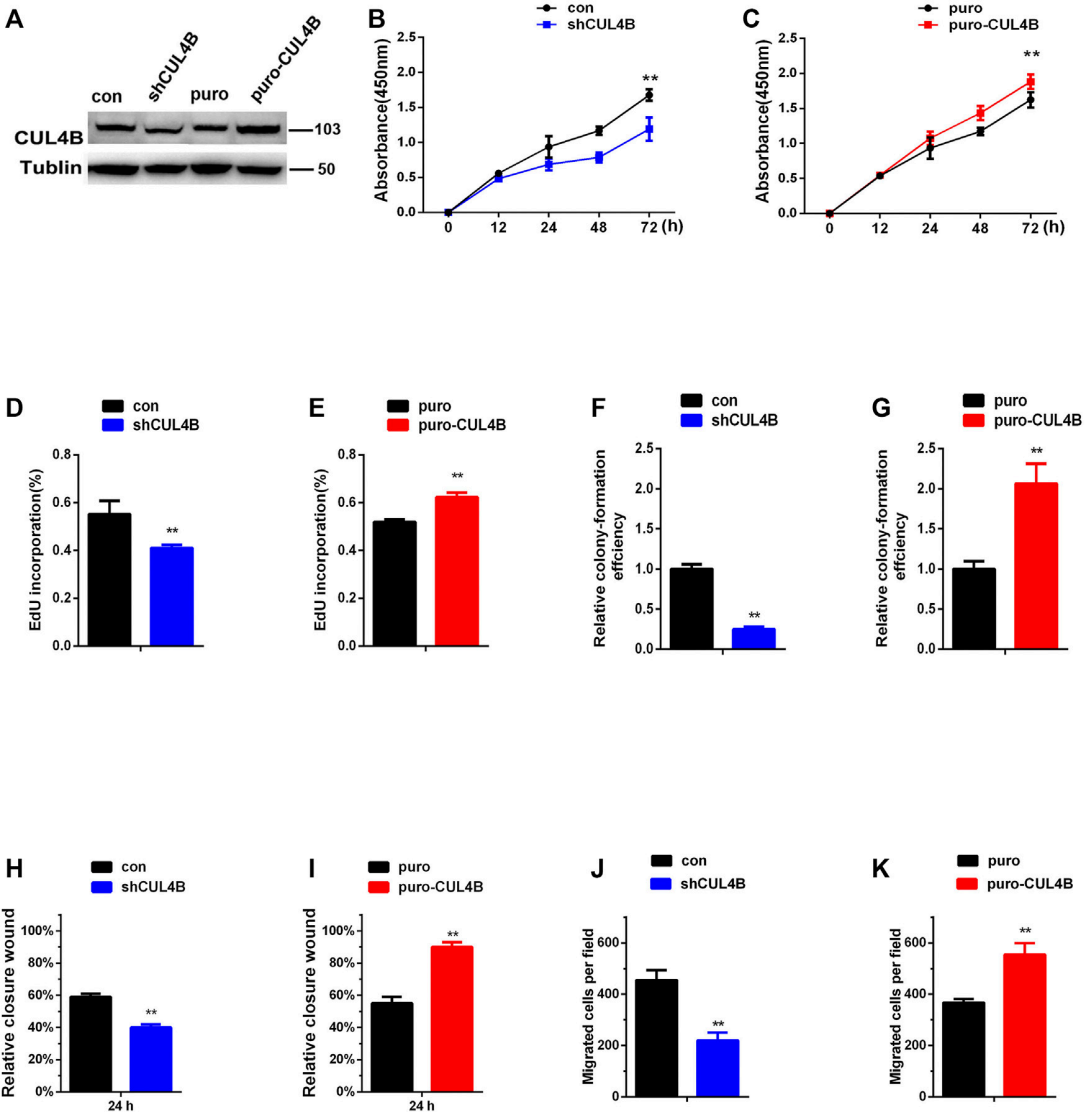
CUL4B promotes the proliferation and migration of PDLSCs. **(A)** Western blot showing knock down and overexpression efficiency of CUL4B in PDLSCs. **(B,C)** Proliferation of PDLSCs with knockdown (shCUL4B) and overexpression of CUL4B (puro-CUL4B) together with corresponding control cells (puro or con), was examined by CCK-8 assays. Quantification of percentage of EdU positive cells in indicated cells was shown in **(D,E)**. Colony formation efficiency of indicated cells was shown in **(F,G)**. The normalized colony number of control cells was set as 1. **(H–K)** Migration ability of indicated cells was examined by wound-healing assay and transwell assay. All quantification analyses were based on independent triplicate experiments. Error bars represent SD. Statistical comparisons were made using two-tailed unpaired t-test, **p < 0.01 compared with negative control.

We began our current study by first assessing the purification of our PDLSCs. As shown in Supplementary Figure S1A, cultured PDLSCs successfully exhibited spindle-shaped morphologies. Then we verified that the isolated PDLSCs were MSCs by analyzing specific MSCs surface markers using flow cytometry. Flow cytometric assays showed that PDLSCs expressed MSCs markers (CD44, CD105 and CD90), but were negative for endothelial cell markers (CD45, CD19, and CD14) (Supplementary Figure S1B).To evaluate the role of CUL4B in PDLSCs, we established PDLSCs with stable knockdown or overexpression of CUL4B (Figure 1A). CCK-8 assays showed that knockdown of CUL4B impeded, while overexpression of CUL4B increased the growth of PDLSCs (Figures 1B,C). An EdU incorporation assays confirmed the role of CUL4B in PDLSCs proliferation (Figures 1D,E and Supplementary Figure S2A,B). The result of colony-forming efficiency assays showed that PDLSCs with stable knockdown of CUL4B displayed much smaller and fewer colonies (Figure 1F and Supplementary Figure S2C). Consistently, overexpression of CUL4B displayed much bigger and more colonies (Figure 1G and Supplementary Figure S2D). Next, we investigated the functions of CUL4B in the migration of PDLSCs. Wound-healing and transwell assays showed that knockdown of CUL4B caused a significant decrease in PDLSC migration (Figures 1H,J and Supplementary Figure S2E,G). In contrast, overexpression of CUL4B led to significantly increase the migration of PDLSCs (Figures 1I,K and Supplementary Figure S2F,H). The effect of CUL4B on PDLSCs apoptosis was analyzed by flow cytometry, and no significant difference between stable CUL4B-knockdown and corresponding control group was observed (Supplementary Figure S2I). Taken together, these results demonstrate that CUL4B promotes proliferation and migration of PDLSCs.

### 3.2 Cullin 4B Enhances Stemness of Periodontal Ligament Stem Cells

**FIGURE 2:**
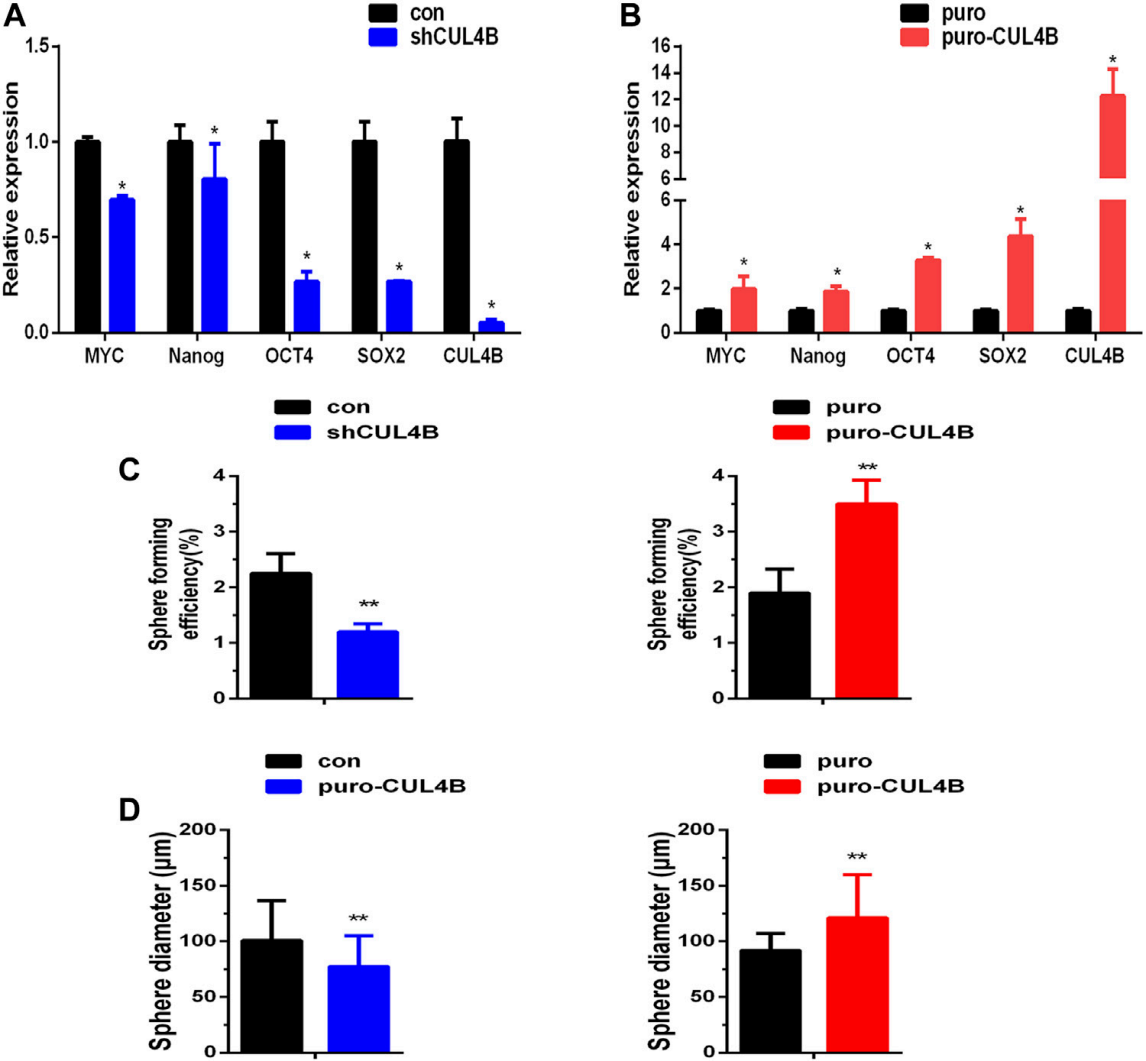
CUL4B enhances stemness of PDLSCs. **(A,B)** Expression levels of stemness markers as indicated in CUL4B stably overexpressing (puro-CUL4B), **(A)** and knockdown [shCUL4B, **(B)**] PDLSCs together with corresponding control cells (puro or con) analyzed by qRT-PCR**. (C,D)** Representative bar graph of the percentages of spheres **(C)** and the diameter of the spheres **(D)** in CUL4B stably overexpressing (puro-CUL4B) and knockdown (shCUL4B) PDLSCs together with corresponding control cells (puro or con). Scale bars: 200 μm. All quantification analyses were based on independent triplicate experiments. Error bars represent SD. Statistical comparisons were made using a two-tailed unpaired t-test, *p < 0.05, **p < 0.01 compared with the negative control.

CUL4B has been reported to enhance the enrichment of cancer stem cells with stem cell-related characteristics (Li et al., 2020; Liu et al., 2020), suggesting that CUL4B may be involved in the stemness of PDLSCs. To investigate the potential interaction of CUL4B with the stemness of PDLSCs, the mRNA expression levels of stemness markers were detected in PDLSCs with stable knockdown and overexpression of CLU4B. The results showed that the stemness markers MYC, Nanog, OCT4 and SOX2 were significantly reduced in stable CUL4B-knockdown PDLSCs whereas increased in CUL4B-overexpressed PDLSCs (Figures 2A,B). Sphere formation assay, commonly used to identify cancer stem cells, was carried out and showed that the size and number of spheres were less and smaller in PDLSCs with knockdown of CUL4B, whereas were more and bigger in CUL4B overexpressed PDLSCs when comparing to the corresponding control group (Figures 2C,D and Supplementary Figure S2J). Taken these data together, it suggests CUL4B plays a key role in regulation of the stemness of PDLSCs.

### 3.3 Cullin 4B Regulates the Osteogenic Differentiation of Periodontal Ligament Stem Cells by Post-transcriptionally Controlling the Expression of RUNX2 Protein

**FIGURE 3:**
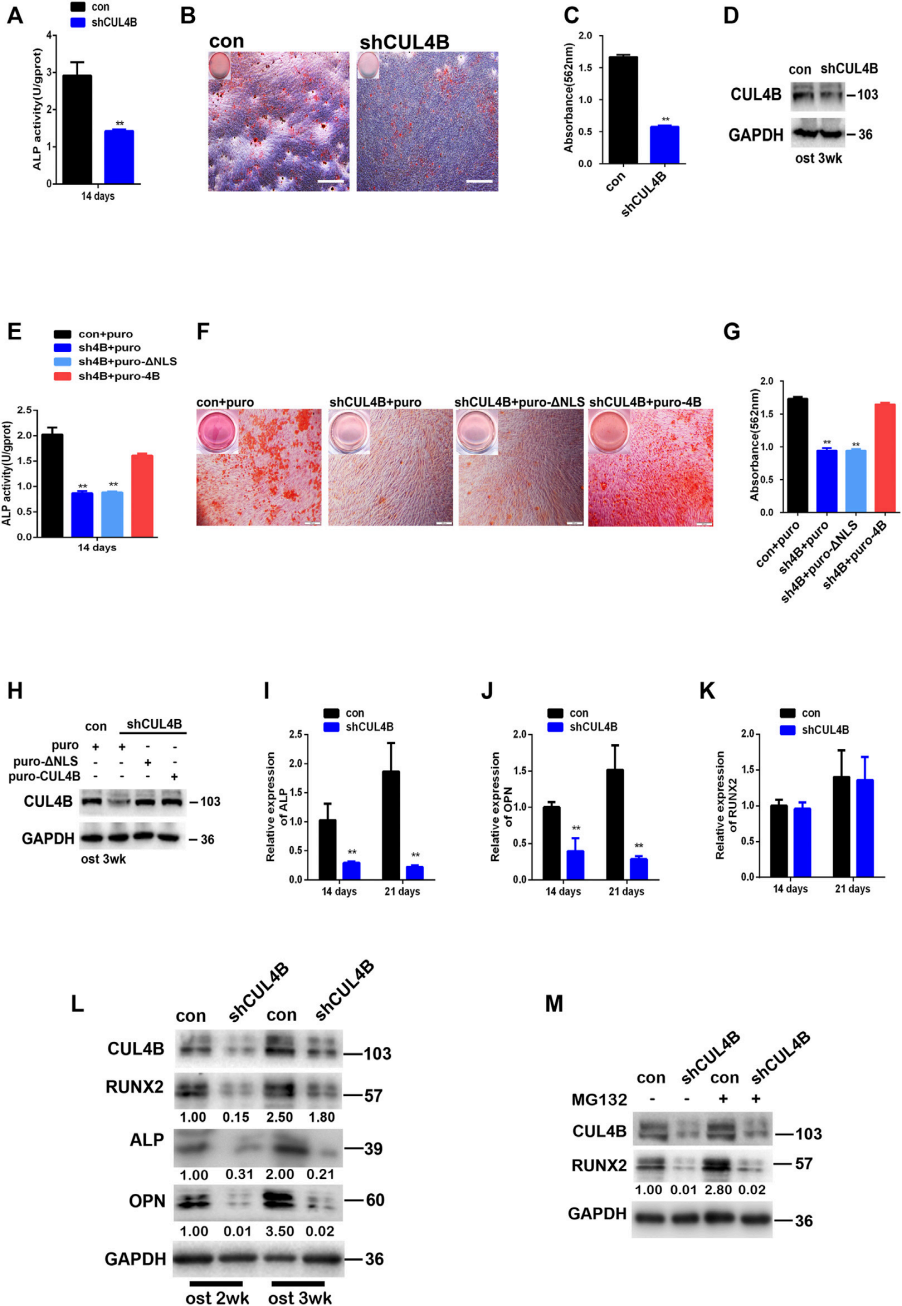
Knockdown of CUL4B suppresses the osteogenic differentiation of PDLSCs through post-transcriptional regulation of RUNX2 protein. **(A)** CUL4B stable knockdown (shCUL4B) and corresponding control (con) PDLSCs were cultured in osteogenic induction medium and were collected at 14 days to measure ALP activity. **(B)** PDLSCs with stable CUL4B knockdown (shCUL4B) or corresponding control vector (con) were cultured in osteogenic induction medium for 21 days, and Alizarin Red staining was performed, scale bars: 200 μm. **(C)** 10% CPC was added, and the concentration of calcium deposition was quantified by absorbance at 562 nm. **(D)** Western blot showing knock down efficiency of CUL4B in PDLSCs after 3 weeks osteogenic induction-f CUL4B stable knockdown (shCUL4B) and corresponding control (con) PDLSCs were infected with indicated CUL4B expressing lentiviral or empty control lentiviral, indicated PDLSCs were cultured in osteogenic induction medium and were collected at 14 days to measure ALP activity **(E)**, for 21 days, Alizarin Red staining was performed **(F)**, 10% CPC was added and the concentration of calcium deposition was quantified by absorbance at 562 nm **(G)**. Western blot showing expression efficiency of indicated CUL4B in PDLSCs after 3 weeks osteogenic induction **(I–K)** CUL4B stable knockdown (shCUL4B) and corresponding control (con) PDLSCs were cultured in osteogenic induction medium for 14 and 21 days and the expression of osteogenesis-related genes were analyzed by qRT-PCR. **(L)** Protein levels of osteogenesis-related genes were determined by western blot in CUL4B stable knockdown (shCUL4B) and in corresponding control (con) PDLSCs after culture in osteogenic induction medium for 14 and 21 days. **(M)** Protein expression levels of RUNX2 in CUL4B stable knockdown (shCUL4B) and in the corresponding control (con) PDLSCs treated with MG132 were determined by western blot. Band intensities underneath the gel images in **(L**,**M)** were measured using ImageJ software (NIH, Bethesda, MD) and are presented as fold change. All quantification analyses were based on independent triplicate experiments. Error bars represent SD. Statistical comparisons were made using a two-tailed unpaired t-test, *p < 0.05, **p < 0.01 compared with the negative control.

Patients with CUL4B mutations manifest disruptions of skeletal development implying that CUL4B might be involved in the formation of mineralized bone, and that osteogenic differentiation is the crucial stage of new bone formation. Since the potential association of CUL4B with the stemness of PDLSCs as shown in above results, we expected that CUL4B might be involved in the regulating osteogenic differentiation of PDLSCs. To test whether CUL4B regulates the osteogenic differentiation of PDLSCs, CUL4B-knockdown PDLSCs were cultivated in osteogenic induction medium to induce osteogenic differentiation. ALP activity has been widely used as a marker of the early osteogenic differentiation of stem cells. In our study, ALP activity was measured after 14 days induction of osteogenic differentiation (Figure 3A). The results showed that the knockdown of CUL4B significantly inhibited ALP activity compared with the control group (con). Extracellular matrix calcification was determined by Alizarin Red S staining and the relative amount of calcium was quantified. The results indicate that after induction, knockdown of CUL4B significantly inhibited mineralization compared with the control group (con) (Figures 3B–D). To further confirm the functional connection between CUL4B and osteogenic differentiation, we performed rescue experiment by transfecting of wild type and nuclear localization signal (NLS)-deleted CUL4B expression vectors into CUL4B knockdown PDLSCs. As shown in Figures 3E–H, after induction of osteogenic differentiation, transfection of wild-type, but not control or nuclear localization signal (NLS)-deleted, CUL4B expression vectors rescued the attenuated ALP activity and mineralization caused by CUL4B knockdown. Together, these data confirmed that CUL4B positively regulate osteogenic differentiation of PDLSCs.

Moreover, the effect of CUL4B on the osteogenic differentiation of PDLSCs was further determined by evaluating genes associated with osteogenic differentiation. The expression levels of osteogenesis-related genes were analyzed by qRT-PCR, we found that the knockdown of CUL4B significantly inhibits the expression levels of genes associated with osteogenic differentiation in PDLSCs, including ALP and OPN (Figures 3I,J). Interestingly, the RUNX2 mRNA expression level, which controls the expression of OPN and ALP by binding to the cis-acting element of their promoter regions was not significantly changed (Figure 3K) (Ge et al., 2007; Bruderer et al., 2014). However, the RUNX2 protein level, together with its targets ALP and OPN, was significantly decreased in CUL4B knockdown PDLSCs after the induction of osteogenesis (Figure 3L). These data suggested that CUL4B decreased the RUNX2 protein expression level not through the transcriptional regulation.

We and others have recently demonstrated that CUL4B regulates the expression of several tumor suppressors, including MYCN, PIK3CA, HER2, SOX4, C-MYC and CDH2 at the posttranscriptional level (Mi et al., 2017; Qi et al., 2018; Qi et al., 2019; Zhao et al., 2019; Li et al., 2020; Liu et al., 2020). Here we found that the RUNX2 mRNA expression level was not significantly changed in CUL4B- knockdown PDLSCs indicating that CUL4B likely regulates RUNX2 at the posttranscriptional level. To confirm this, we treated CUL4B-knockdown cells with MG132 and observed that MG132 did not block the reduction of RUNX2 protein (Figure 3M), which suggests that CUL4B does not regulate the degradation of RUNX2. Taking these data together, we concluded that CUL4B regulates the expression of RUNX2 protein at the post-transcriptional level in PDLSCs.

### 3.4 Cullin 4B Upregulates RUNX2 by Repressing the Expression of miR-320c/miR-372/373-3p

**FIGURE 4:**
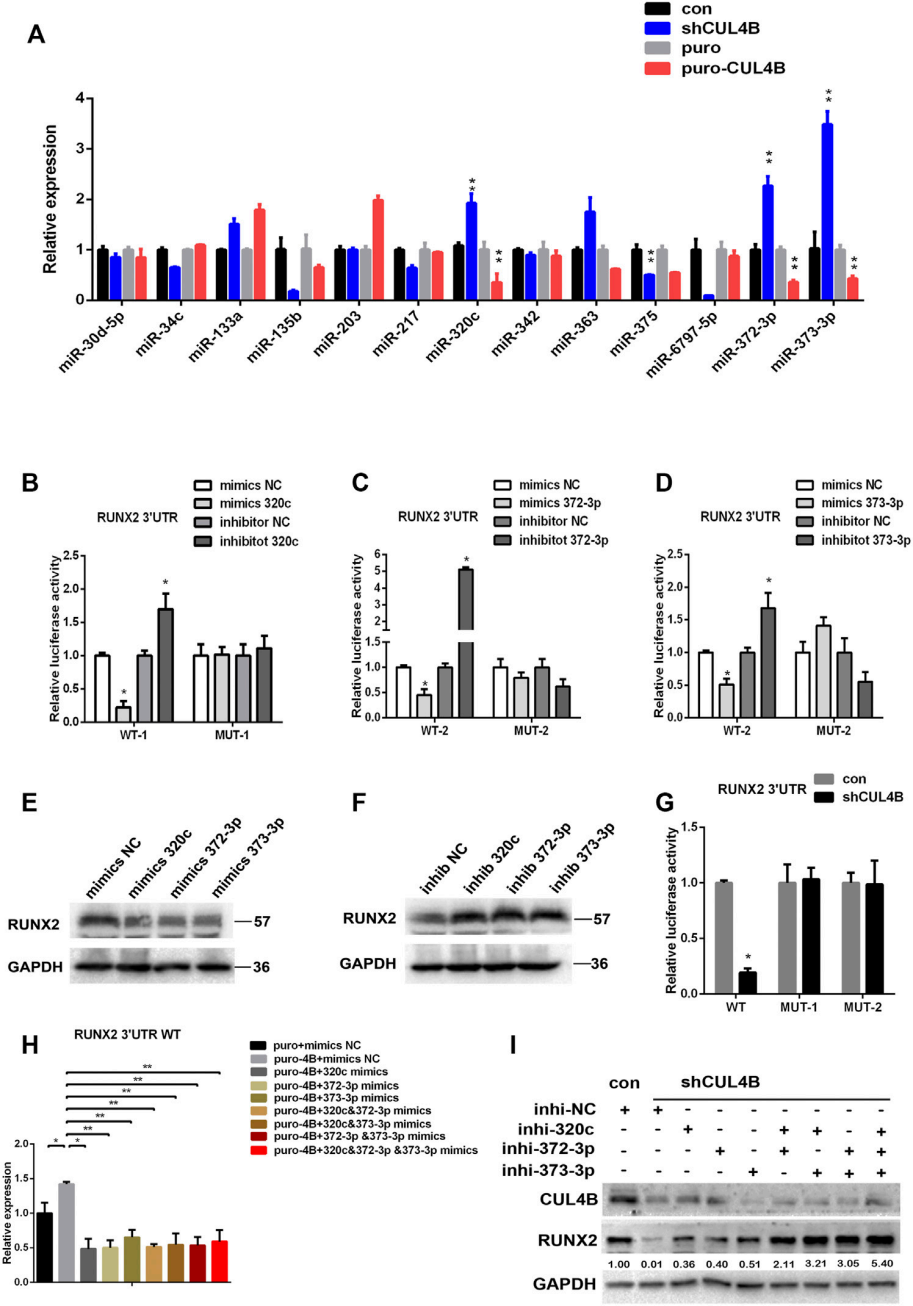
CUL4B upregulates RUNX2 by repressing the expression of miR-320c/miR-371–373. **(A)** Expression of candidate miRNAs in CUL4B stable overexpressing (puro-CUL4B) or knockdown (shCUL4B) PDLSCs together with the corresponding control cells (puro or con) were determined by qRT-PCR. **(B–D)** Luciferase reporter assays of PDLSCs, which were transiently transfected with the wild-type construct of pmirGLO-RUNX2 3′-UTR (WT), with mutant constructs of pmirGLO-RUNX2 3′-UTR (MUT-1: mutated miR-320c binding site, MUT-2: mutated miR-372/3-3p binding site), with control RNA (NC) or with miR-320c and miR-372-3p/373-3p mimics. **(E–F)** Western blot analysis of RUNX2 expression in PDLSCs transfected with miR-320c and miR-372-3p/373-3p mimics or inhibitors and relative control RNAs (Mimics-NC or Inhibitor NC). **(G)** Luciferase reporter assays of CUL4B knockdown (shCUL4B) or control (con) PDLSCs that were transiently transfected with vectors carrying wild-type RUNX2 3′UTR (WT) or mutant RUNX2 3′UTR (MUT-1 or MUT-2 as above). **(H)** Luciferase reporter assays showing decreased luciferase activity of wild-type CUL4B overexpressing PDLSCs (puro-CUL4B) that was rescued by miR-320c and miR-372-3p/373-3p. **(I)** Western blotting analysis showing decreased RUNX2 expression in CUL4B knockdown PDLSCs (shCUL4B) was rescued by 320c and miR-372-3p/373-3p repression. Band intensities underneath the gel images in **(I)** were measured using ImageJ software (NIH, Bethesda, MD) and are presented as fold change. All quantification analyses were based on independent triplicate experiments. Error bars represent SD. Statistical comparisons were made using a two-tailed unpaired t-test, *p < 0.05, **p < 0.01 compared with the negative control.

Next, we investigated the underlying molecular mechanisms by which CUL4B controls the protein level of RUNX2 in PDLSCs. Based on a previous report and our findings above, we hypothesized that miRNAs might be involved in the upregulation of RUNX2 by CUL4B. It has been reported that several miRNAs regulate RUNX2 (Narayanan et al., 2019), so we first asked whether those miRNAs were regulated by CUL4B and could subsequently modulate the expression of RUNX2. We focused on 11 miRNAs that have been reported to target RUNX2 (Supplementary Table S1). Meanwhile, CUL4B has been shown to epigenetically represses several miRNAs (Zou et al., 2013; Mi et al., 2017; Qi et al., 2018; Zhao et al., 2019; Li et al., 2020; Liu et al., 2020), so we determined if these miRNAs regulated by CUL4B could potentially bind RUNX2 3′-untranslated region (UTR) to modulate RUNX2 expression. We used three different bioinformatic prediction tools to analyze 9 miRNAs or miRNA clusters that are directly targeted by CUL4B (Supplementary Table S2) and found that miR-372/373-3P could potentially bind the RUNX2 3′-UTR. So, we selected miR-372/373-3P and 11 other miRNAs which regulate RUNX2 as candidates. To evaluate the potential roles of these miRNAs in RUNX2 protein expression we first examined the expression of these candidates’ miRNAs in PDLSCs with knockdown or overexpression of CUL4B. Among them, miR-320c and miR-372/373-3p were found to be consistently changed with the knockdown or overexpression of CUL4B (Figure 4A). Therefore miR-320c and miR-372/373-3p were selected for further investigation.

To determine the role of miR-320c and miR-372/373-3p in regulating RUNX2 expression, we subcloned the RUNX2 3′-UTR with or without mutated miR-320c and miR-372/373-3p binding sites into the luciferase reporter vector pmir-GLO (Supplementary Figure S3A,B). As shown in Figures 4B–D, luciferase reporter assays showed that while overexpression of miR-320c and miR-372/3-3p in PDLSCs significantly repressed the relative luciferase activity of the wild-type (WT) construct of pmirGLO-RUNX2 3′-UTR, it had little effect on the activity of the construct of pmirGLO-RUNX2 3′-UTR containing the mutated miR-320c (MUT-1) or miR-372/3-3p (MUT2) binding sites. In addition, we showed that miR-320c and miR-372/373-3p mimics reduced RUNX2 protein expression, while miR-320c or miR-372/373-3p inhibitors resulted in increased amounts of RUNX2 protein (Figures 4E,F). Together, our data demonstrate that miR-320c and/or miR-372/373-3p can repress RUNX2 expression in PDLSCs.

We next investigated whether CUL4B regulating RUNX2 expression in PDLSCs is mediated by miR-320c and/or by miR-372/373-3p. Luciferase reporter assays showed that the activity of the construct containing miR-320c and miR-372/3-3p binding site of RUNX2 3′UTR but not the construct containing miR-320c and miR-372/3-3p mutant binding site of RUNX2 3′UTR was repressed by knockdown of CUL4B (Figure 4G), and transfection with miR-320c or miR-372/373-3p mimics impeded the increase in the WT construct activity caused by high expression of CUL4B (Figure 4H). Furthermore, the downregulation of RUNX2 by CUL4B knockdown could be efficiently attenuated by transfection with inhibitors of miR-320c or miR-372-3p/373-3p (Figure 4I). Taking these data together, we demonstrated that CUL4B controls the expression of RUNX2 mediated by miR-320c and miR-372/373-3p in PDLSCs.

### 3.5 Cullin 4B Epigenetically Represses the Expression of miR-320c/miR-372-3p/373-3p in Periodontal Ligament Stem Cells

**FIGURE 5:**
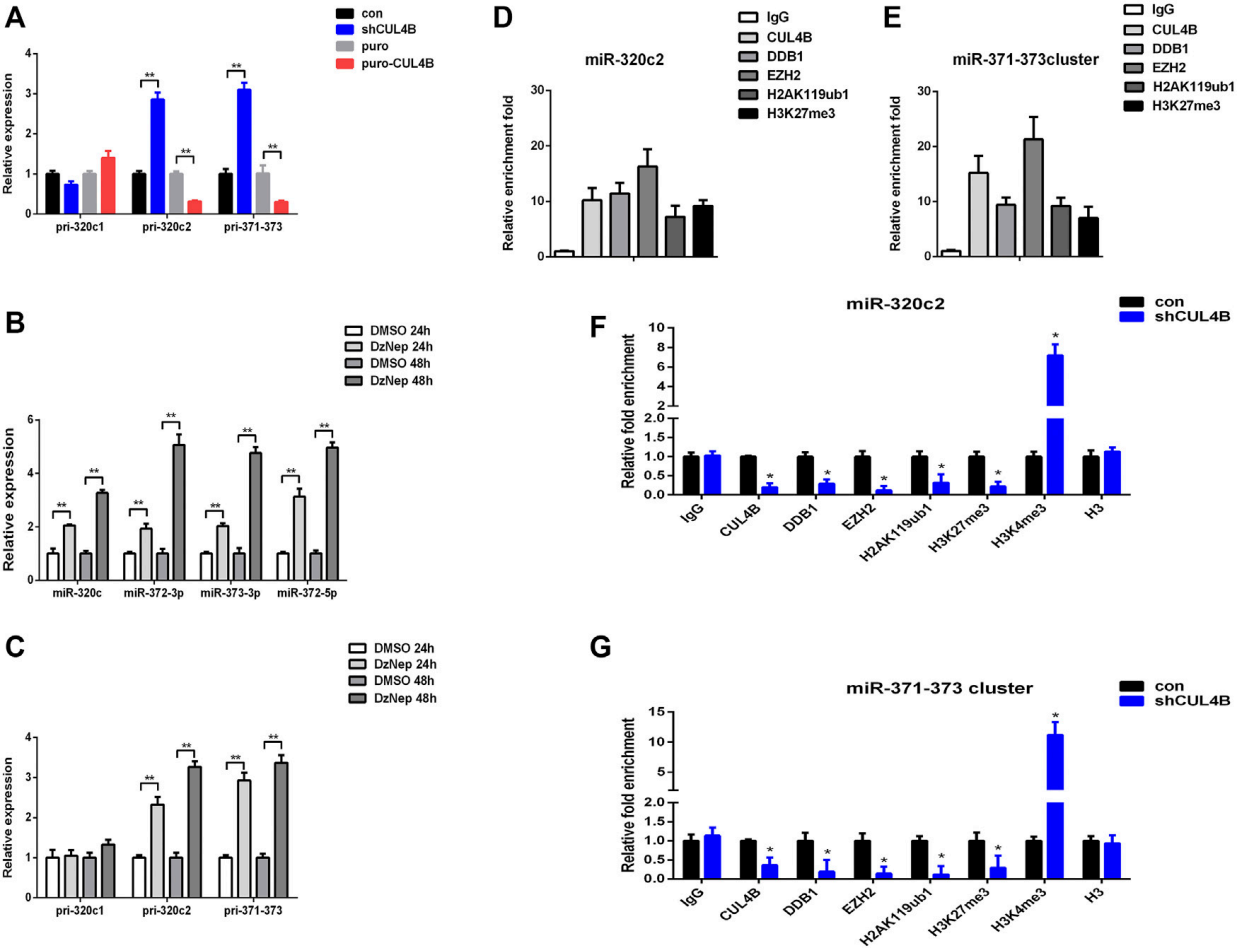
CUL4B epigenetically represses the expression of miR-320c/miR-371–373 in PDLSCs. **(A)** pri-miR levels in CUL4B stable overexpressing (puro-CUL4B) and knockdown (shCUL4B) PDLSCs together with the corresponding control cells (con or puro) were analyzed by qRT-PCR. **(B,C)** miRNA and pri-miRNA levels in PDLSCs treated with 1 µM DzNep for 24 and 48 h determined by qRT-PCR. **(D,E)** ChIP assays performed using PDLSCs with antibodies against IgG, CUL4B, DDB1, EZH2, H2AK119ub and H3K27me3. qRT-PCR was performed with primers at the region of miR-320c **(D)** and miR-371–373 **(E)** promoter. **(F,G)** ChIP assays performed in CUL4B stable knockdown (shCUL4B) PDLSCs together with the corresponding control cells (con) with antibodies against CUL4B, DDB1, EZH2, H2AK119ub, H3K27me3, H3K4me3, and Histone H3. qRT-PCR was performed with primers at the region of miR-320c **(F)** and miR-371–373 **(G)** promoters. All quantification analyses were based on independent triplicate experiments. Error bars represent SD. Statistical comparisons were made using a two-tailed unpaired t-test, *p < 0.05, **p < 0.01 compared with the negative control.

We then characterized how CUL4B regulates the function of miR-320c and miR-372-3p/373-3p in PDLSCs. Mature miR-320c is derived from miR-320c1 and miR-320c2 on chromosome 18, and miR-372-3p/373-3p, miR-371a, miR-371b and miR-372/373-5p all belong to the miR-371–373 cluster. We first analyzed the expression levels of primary polycistronic miRNA transcript (pri-miRNA) of miR-320c and miR-371–373 cluster. Pri-miR-320c2 and pri-miR-371–373 levels were remarkably raised but not pri-miR-320c1 in CUL4B stable knockdown PDLCSs but were reduced in CUL4B-overexpressing PDLSCs (Figure 5A), which suggests that CUL4B may repress the initial step of miR-320c and miR-372/373 biogenesis. A previous study showed that EZH2 binds to the promoter region of miR-320c and miR-372-3p/373-3p (Alzrigat et al., 2017; Liu et al., 2020). We then measured the expression of mature miR-320c and other members of the miR-371–373 cluster: miR-372-3p and miR-373-3p and miR-372-5p in PDLSCs treated with 1 µM EZH2 inhibitor DzNep for 24 and 48 h, using DMSO treatment as a control. These mature miRNAs were significantly upregulated after treatment with DzNep. Consistent with that, pri-miR-320c2 and pri-miR-371–373 expression levels were also significantly raised but not pri-miR-320c1 after treated with DzNep (Figures 5B,C). We then used Chromatin immunoprecipitation (ChIP) assays with primer pairs covering those regions as reported before. CUL4B, EZH2 and DDB1 co-occupancy was detected at those regions and was associated with enriched H2AK119ub1 and H3K27me3 in PDLSCs (Figures 5D,E). Knockdown of CUL4B caused a significant reduction in the recruitment of CUL4B, EZH2 and DDB1, consequently decreased H2AK119ub1, H3K27me3 at miR-320c2 and miR-371–373 promoter, and increased H3K4me3 at miR-320c2 and miR-371–373 promoter (Figures 5F,G). Taken together, our data suggested that CUL4B functions to repress miR-320c2 and miR-371–373 transcription in PDLSCs by promoting H2AK119 monoubiquitination and consequently the recruitment of the PRC2 complex.

### 3.6 Cullin 4B Regulates the Osteogenic Differentiation of Periodontal Ligament Stem Cells *via* the miR-320c and miR-372-3p/373-3p-RUNX2 Axis

**FIGURE 6:**
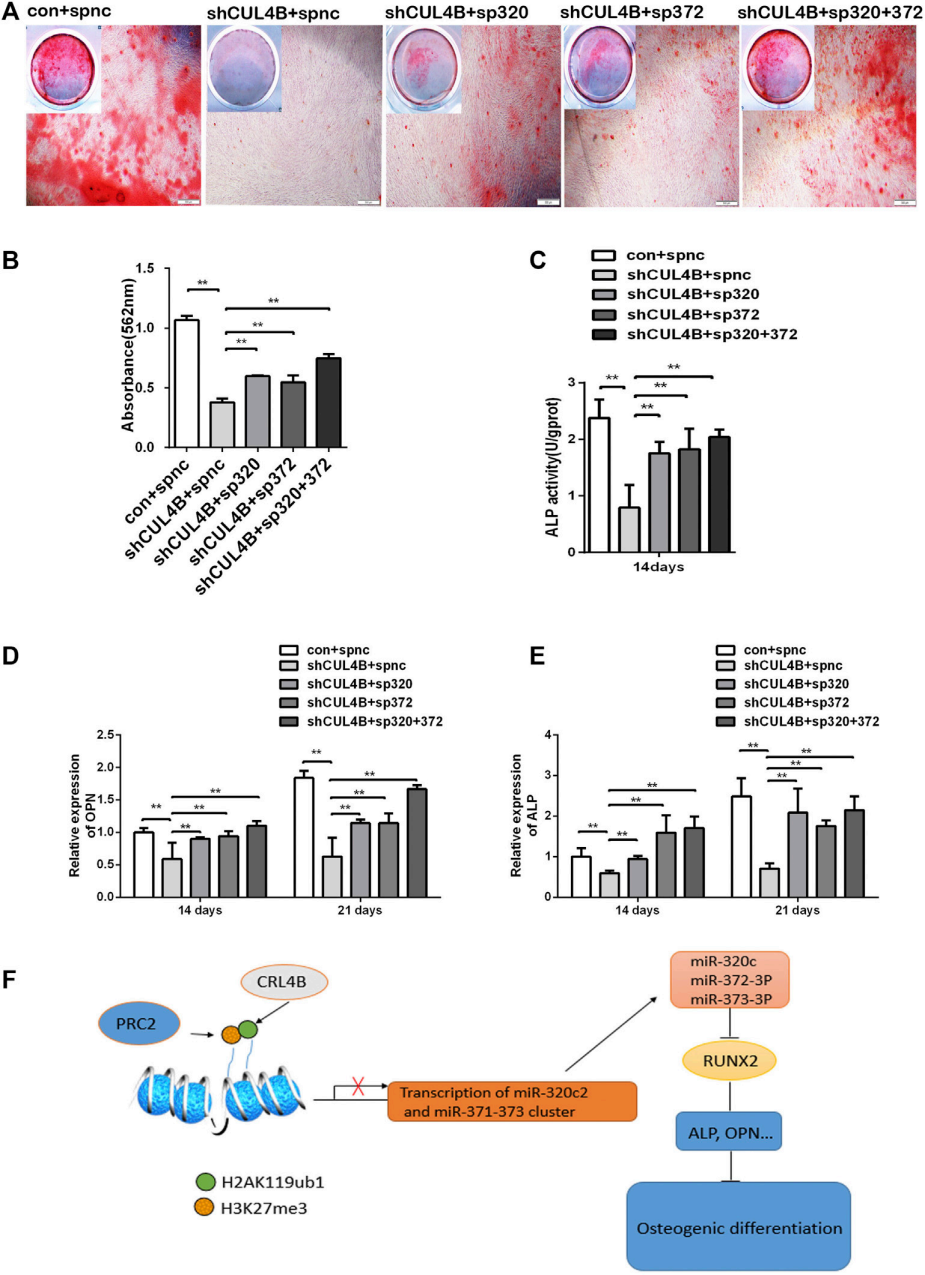
CUL4B regulates the osteogenic differentiation of PDLSCs via the miR-320c and miR-372-3p/373-3p-RUNX2 axis. **(A–E)** CUL4B stable knockdown (shCUL4B) or control (con) PDLSCs were infected with lentivirus carrying: miR-320c sponge (sp320c), miR-372-3p/373-3p sponge (sp372) or control sponge (spnc), after which the infected cells were cultured in osteogenic induction medium for 14 or 21 days. At 21 days, the cells were collected for Alizarin Red staining **(A)** and analysis of the concentration of calcium deposition **(B)**. Scale bars: 200 μm. **(C)** ALP activity of different groups of PDLSCs were analyzed at day 14.The expression of osteogenesis-related genes OPN **(D)** and ALP **(E)** in different groups of PDLSCs collected at day 14 were determined by qRT-PCR. **(F)** Schematic of the CUL4B-miR-320c and miR-372/373-RUNX2 axis and its role in the regulation of PDLSC osteogenesis. All quantification analyses were based on independent triplicate experiments. Error bars represent SD. Statistical comparisons were made using a two-tailed unpaired t-test, *p < 0.05, **p < 0.01 compared with the negative control.

Finally, the role of the miR-320c and miR-372-3p/373-3p-RUNX2 axis in the osteogenic differentiation of PDLSCs regulated by CUL4B was further evaluated by gene knockdown experiments using lentiviral-mediated stable expression of miRNA sponges. The expression levels of miR-320c and miR-372-3p/373-3p were significantly decreased in PDLSCs infected with the lentivirus expression of miR-320c and miR-372-3p sponges (Supplementary Figure S4A–C). As shown by Alizarin Red staining and ALP activity assay (Figures 6A–C), the downregulation of osteogenic activities by CUL4B RNAi could be efficiently attenuated by inhibitors of miR-320c and miR-372-3p/373-3p after the lentiviral-mediated stable expression of miRNA sponges.

The decreased expression of the osteoblast marker genes ALP and OPN by CUL4B knockdown could also be efficiently attenuated in PDLSCs by the stable expression of miRNA sponges (Figures 6D,E). Taken together, these data implied that the miR-320c and miR-372-3p/373-3p-RUNX2 axis is implicated in modulating the osteogenic differentiation of PDLSCs regulated by CUL4B.

In summary, the CRL4B complex can epigenetically repress the transcription of miR-320c and miR-372/373-3p, which directly targets RUNX2, and consequently activates the osteoblast marker genes ALP and OPN modulating osteogenic differentiation (shown schematically in Figure 6F).

## 4 Discussion

PDLSCs, a subtype of MSCs, are an excellent source of cells with self-renewing capability and multidirectional differentiation potential for use in periodontal tissue engineering and bone regenerative applications (Seo et al., 2004; Tassi et al., 2017). Understanding the mechanisms of self-renewal and multi-directional differentiation is of great significance for the application of this type of cell. PDLSCs derived from dental tissues have the advantages of availability, rapid culture expansion and hypoimmunogenicity, not only are widely used as favorable seed cells for periodontal tissues and bone regeneration but also as a suitable cell model to study the self-renewing ability and multidirectional differentiation mechanisms, especially osteogenic differentiation.

Cullin 4B (CUL4B), which assembles the CUL4B-RING ubiquitin ligase (CRL4B) complex, participates in a variety of developmental and physiological processes. CUL4B plays a critical role in regulating DNA replication and cell-cycle progression (Zou et al., 2009; Zou et al., 2013). Previous studies have indicated that CUL4B exerts biological functions in various types of cancers (Hu et al., 2012; Mi et al., 2017; Qi et al., 2018; Jiao et al., 2019; Qi et al., 2019; Li et al., 2020). Mutations in human CUL4B cause intellectual disability (Tarpey et al., 2007; Zou et al., 2007) and patients with CUL4B mutations manifest disruptions in skeletal development such as a short stature and brachydactyly (Badura-Stronka et al., 2010; Isidor et al., 2010; Kerzendorfer et al., 2011; Ravn et al., 2012). Bone formation is the basis of skeletal development, which is associated with osteogenic differentiation. But how CUL4B manifests skeletal development or bone formation is still poorly defined. So, given the advantages of PDLSCs mentioned above, in this study we induced the osteogenic differentiation of PDLSCs as a cell model to investigate how CUL4B contributes to osteogenesis as well as its role in regulating the biological properties of MSCs, which provided insights into how CUL4B manifests skeletal development and bone formation. We found that the lack of CUL4B impedes the proliferation and migration of PDLSCs. CUL4B enhances stemness of PDLSCs. Our silencing experiments also confirmed that reducing the expression of CUL4B led to a significant inhibition of PDLSC osteogenesis. This is the first demonstration that CUL4B is a new regulator of osteogenic differentiation in PDLSCs.

RUNX2, which is a key TF involved in regulating osteogenesis, is a member of the mammalian RUNT related TF family (Bruderer et al., 2014). RUNX2 can upregulate expression of the ColeI, ALP and OPN genes (Ge et al., 2007). The regulation of RUNX2 is pivotal in the osteogenesis of PDLSCs and other types of stem cells. miRNAs are small non-coding RNAs that are 17–25 nucleotides long. miRNAs regulate the expression of target genes at the post-transcriptional level by hybridizing with target mRNAs at the 3′untranslated region (3′UTR), consequently silencing the target genes and thereby controlling the biosynthesis of target proteins (Wahid et al., 2010; Vimalraj and Selvamurugan, 2013). A large number of miRNAs have been shown to directly bind to the 3′UTR of RUNX2. In addition, because RUNX2 has a large 3′UTR (3.777 kb), RUNX2 is likely targeted by several groups of miRNAs. Previous studies have demonstrated that RUNX2 is targeted by miR-320c in human mesenchymal (skeletal) stem cells (Hamam et al., 2014). In the current study, we demonstrated that RUNX2 is indeed a direct target for miR-320c in PDLSCs. We found that RUNX2 is also a direct target of miR-372-3p/373-3p. The results show that miR-320c and miR-372/373-3p repress RUNX2 expression and consequently regulate the osteogenesis of PDLSCs, which suggests that miR-320c and miR-372/373-3p are crucial targets of CUL4B in PDLSCs. CUL4B was reported to negatively regulate the biogenesis of miRNAs in many types of cancer cells. We previously showed that CUL4B functions to repress miRNA transcription, an important step by which CUL4B exerts its functions both in physiology and in pathology (Li et al., 2020; Liu et al., 2020). We demonstrated in this study that CUL4B binds to the promoters of miR-320c2 and the miR-371–373 cluster to catalyze H2AK119 monoubiquitination and consequently the recruitment of the PRC2 complex. Cheng and Zhou have reported that the LPS-induced overexpression of EZH2 suppressed the osteogenic differentiation of PDLSCs under inflammatory conditions (Cheng and Zhou, 2020). Treatment with DzNep restores the osteogenic differentiation defect of endogenous osteoporotic BMSCs (Jing et al., 2016). CUL4B epigenetically represses miR-320c/miR-371–373 in PDLSCs by promoting the monoubiquitination of H2AK119 and the consequent binding of EZH2. Hamam et al. reported that the miR-320c/RUNX2 axis regulates osteogenic differentiation in human mesenchymal cells (Hamam et al., 2014), but the roles of miR-372-3p/373-3p, which is a new regulator of RUNX2 and a target of EZH2 in osteogenic differentiation remain poorly understood. Our results show that CUL4B regulates the expression of RUNX2 protein at the post-transcriptional level. Thus, CUL4B is a new regulator of RUNX2.

CUL4B possesses oncogenic properties in a variety of human cancers (Hu et al., 2012; Yuan et al., 2015; Mi et al., 2017; Qi et al., 2018; Qi et al., 2019; Li et al., 2020; Liu et al., 2020). RUNX2 is often aberrantly reactivated in many cancers. A recent clinical study reported high-expression levels of RUNX2 in tumors derived from epithelial tissues including breast (Chang et al., 2014), pancreas (Kayed et al., 2007), prostate (Lim et al., 2010), lung (Herreño et al., 2019) and colorectal (Ji et al., 2019), in which CUL4B is also aberrantly reactivated. We demonstrated that CUL4B is a new regulator of RUNX2 for osteogenic differentiation in PDLSCs, but the relationship between CUL4B and RUNX2 in cancers remains unknown. Therefore, further investigations are needed to characterize the mutual interactions of CUL4B and RUNX2 in cancers including miR-320c2 and miR-372-3p/373-3p. In addition, in this study we demonstrated the CUL4B-miR-320c and miR-372/373-3p-RUNX2 axis regulates the osteogenic differentiation *in vitro*. However, the CUL4B-miR-320c and miR-372/373-3p-RUNX2 axis that regulate osteogenic differentiation *in vivo* remain largely unclear. It would be interesting to further investigate the signaling activation in animal models.

In summary, CUL4B can epigenetically repress the expression of miR-320c and miR-372/373-3p that directly affects the level of RUNX2, and consequently activates the osteoblast marker genes ALP and OPN (Figure 6F). Intervention of the CUL4B-miR-320c and miR-372/373-RUNX2 axis may have important implications in transplantation therapies of PDLSCs and could be used for periodontal tissue engineering and guiding bone regeneration.

## Data Availability

The original contributions presented in the study are included in the article/Supplementary Material, further inquiries can be directed to the corresponding authors.
