# Supplementary material for: CUL4B Upregulates RUNX2 to Promote the Osteogenic Differentiation of Human Periodontal Ligament Stem Cells by Epigenetically Repressing the Expression of miR-320c and miR-372/373-3p
Source: Front Cell Dev Biol. 2022 Jun 16;10:921663. doi: 10.3389/fcell.2022.921663 (PMC9243338; doi:10.3389/fcell.2022.921663)
Supplement: Supplementary file 1 [file DataSheet1.docx]

**Supplementary information**

**
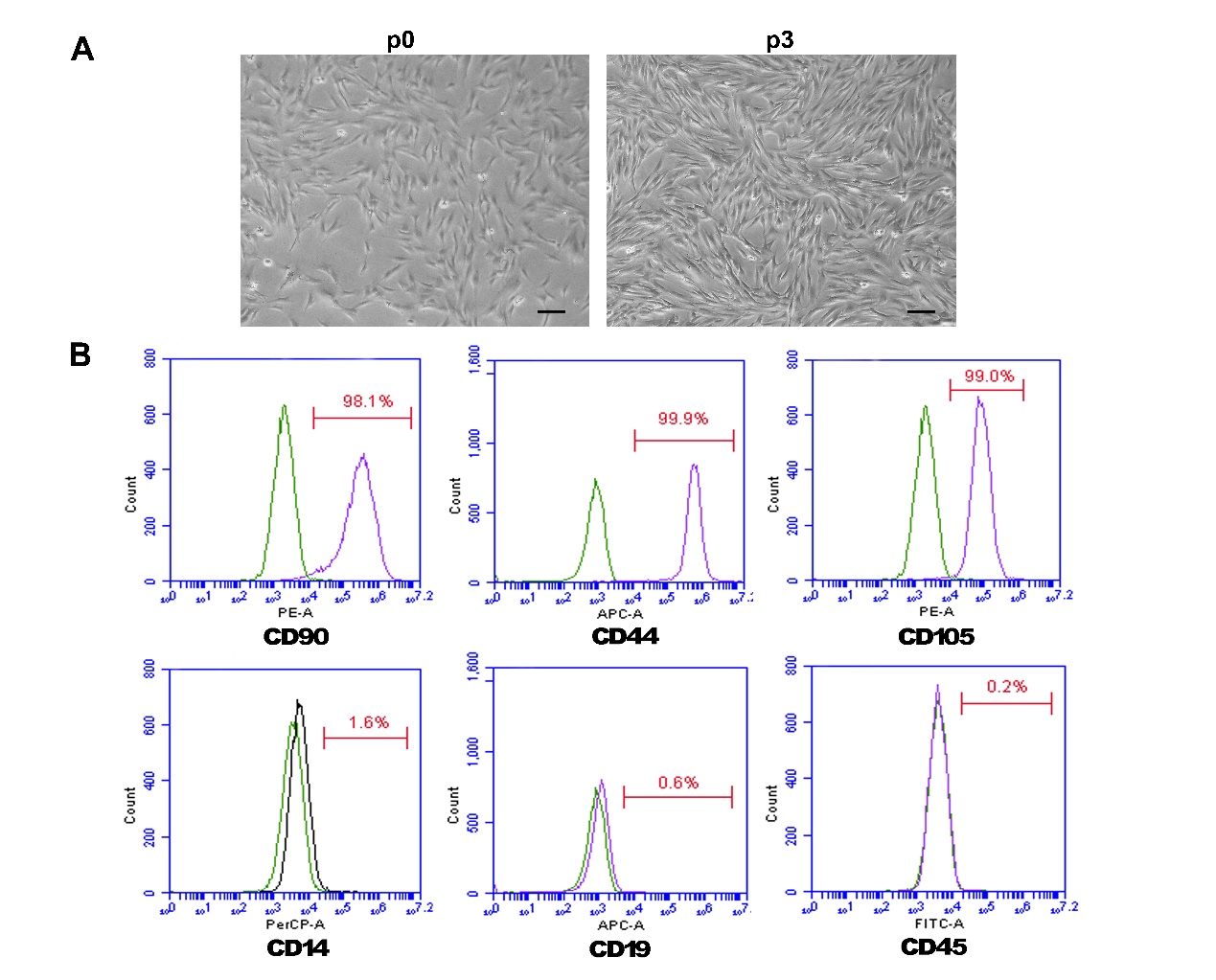
**

**Fig. S1 (A)** Imaging of PDLSCs cultured in normal media before the first (P0) or after the third passage (P3) by optical microscopy. **(B)** Surface markers of PDLSCs were analyzed by flow cytometry (FACS) and were positive for mesenchymal markers (CD90, CD44 and CD105) and negative for endothelial markers (CD14, CD19 and CD45). Scale bars: 200 μm.

**
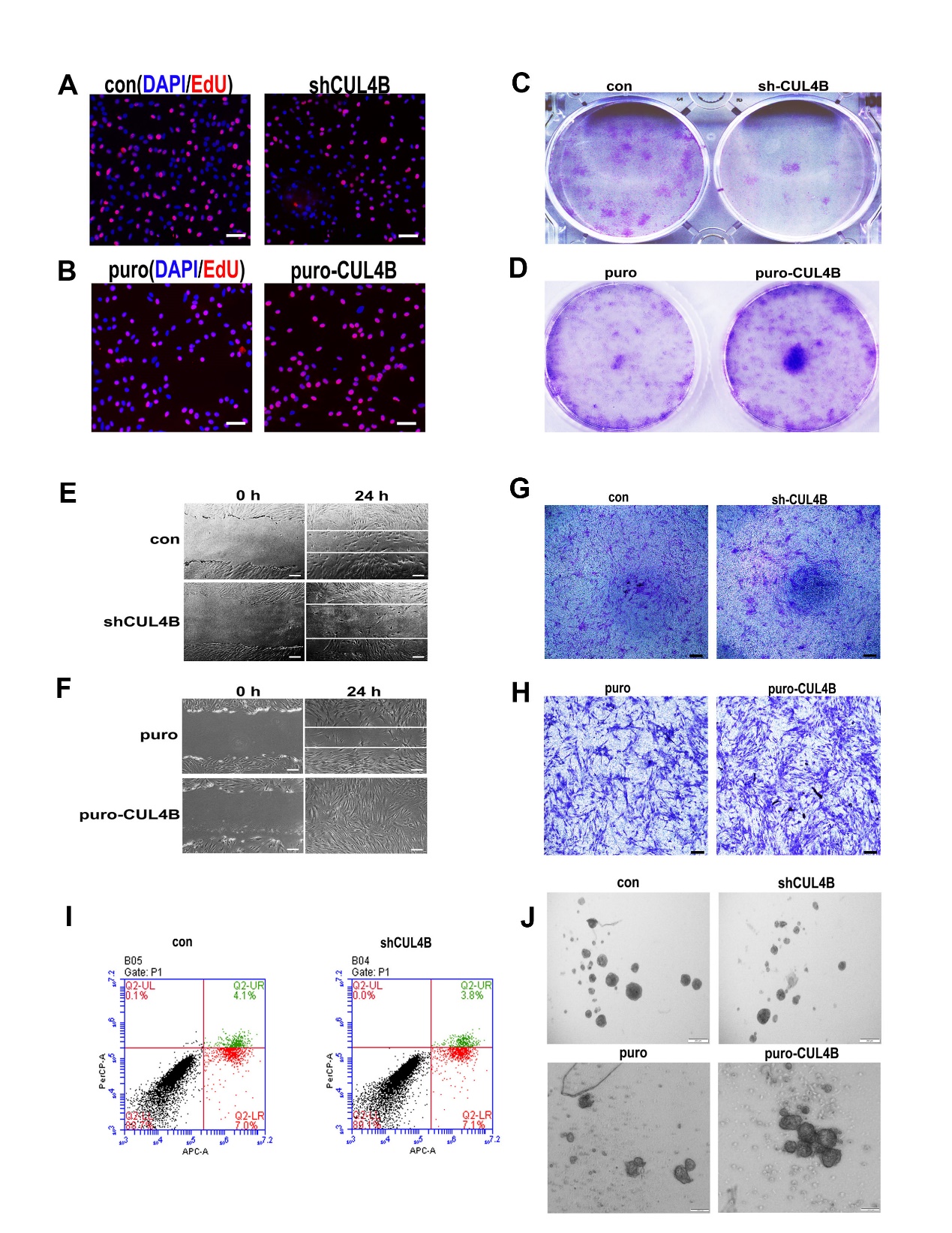
**

**Fig. S2** **(A**, **B)** Representative images of EdU incorporation assays of CUL4B knockdown (shCUL4B) and overexpression of CUL4B (puro-CUL4B) PDLSCs; EdU signals are red, DAPI signals are blue for nuclei. Scale bars: 100 μm. The images of colony formation were shown in **(C**, **D)**. **(E**, **F)** Representative images of wound-healing assays of CUL4B knockdown (shCUL4B) and overexpression of CUL4B (puro-CUL4B) PDLSCs were shown. **(G**, **H)** Representative images of transwell migration assays of CUL4B knockdown (shCUL4B) and overexpression of CUL4B (puro-CUL4B) PDLSCs were shown. **(I)** Effect of CUL4B on PDLSCs apoptosis was determined by flow cytometry using Annexin V-APC and 7-AAD staining. **(J)** Representative images of spheres formation ability evaluation on CUL4B knockdown (shCUL4B) and overexpression of CUL4B (puro-CUL4B) PDLSCs were shown. Scale bars: 200 μm.


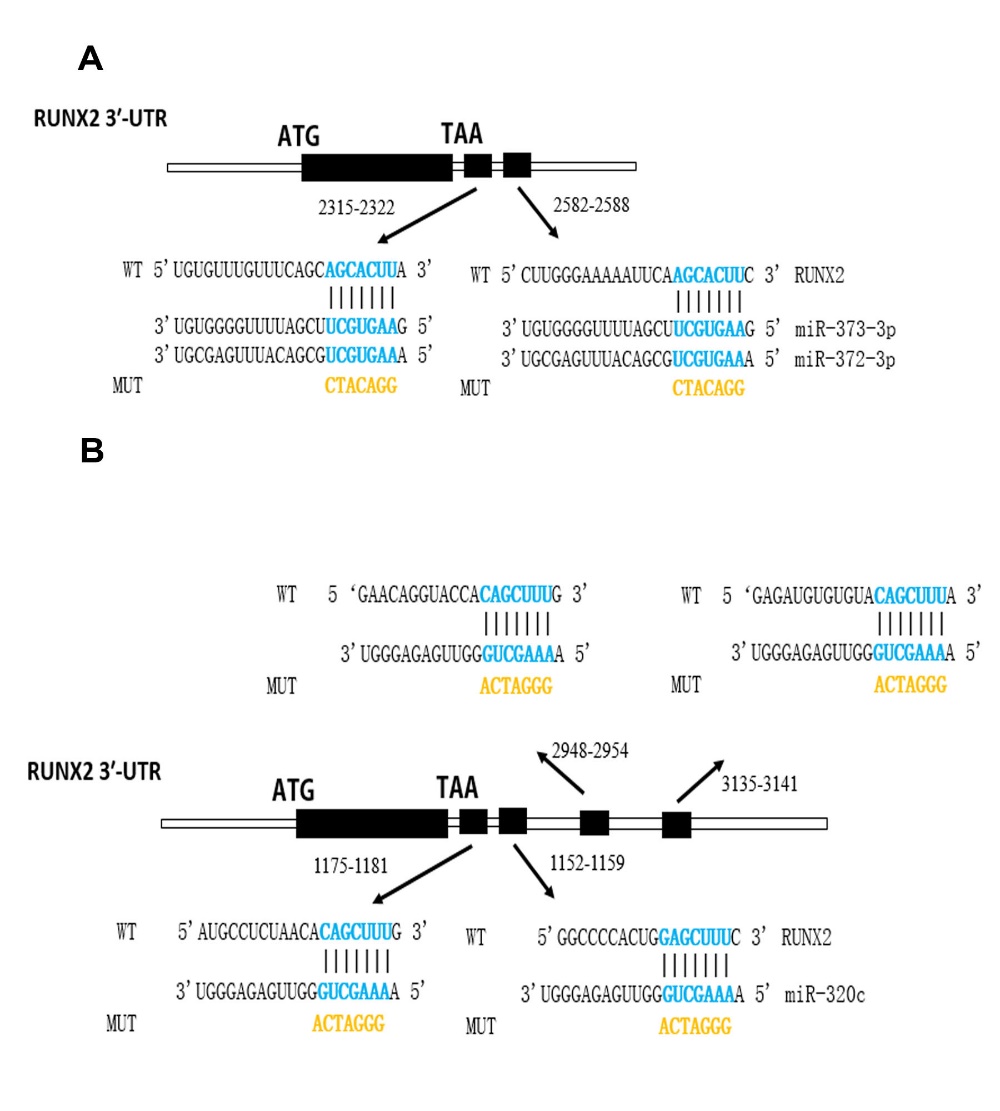


**Fig. S3** Schematic of putative miR-372/373-3p and miR-320c binding sites in the RUNX2 3′UTR. **(A)** Schematic of putative 2 miR-372/373-3p binding sites in the RUNX2 3′UTR (WT: blue, MUT: yellow). **(B)** Schematic of putative 4 miR-320c binding sites in the RUNX2 3′UTR (WT: blue, MUT: yellow).

**
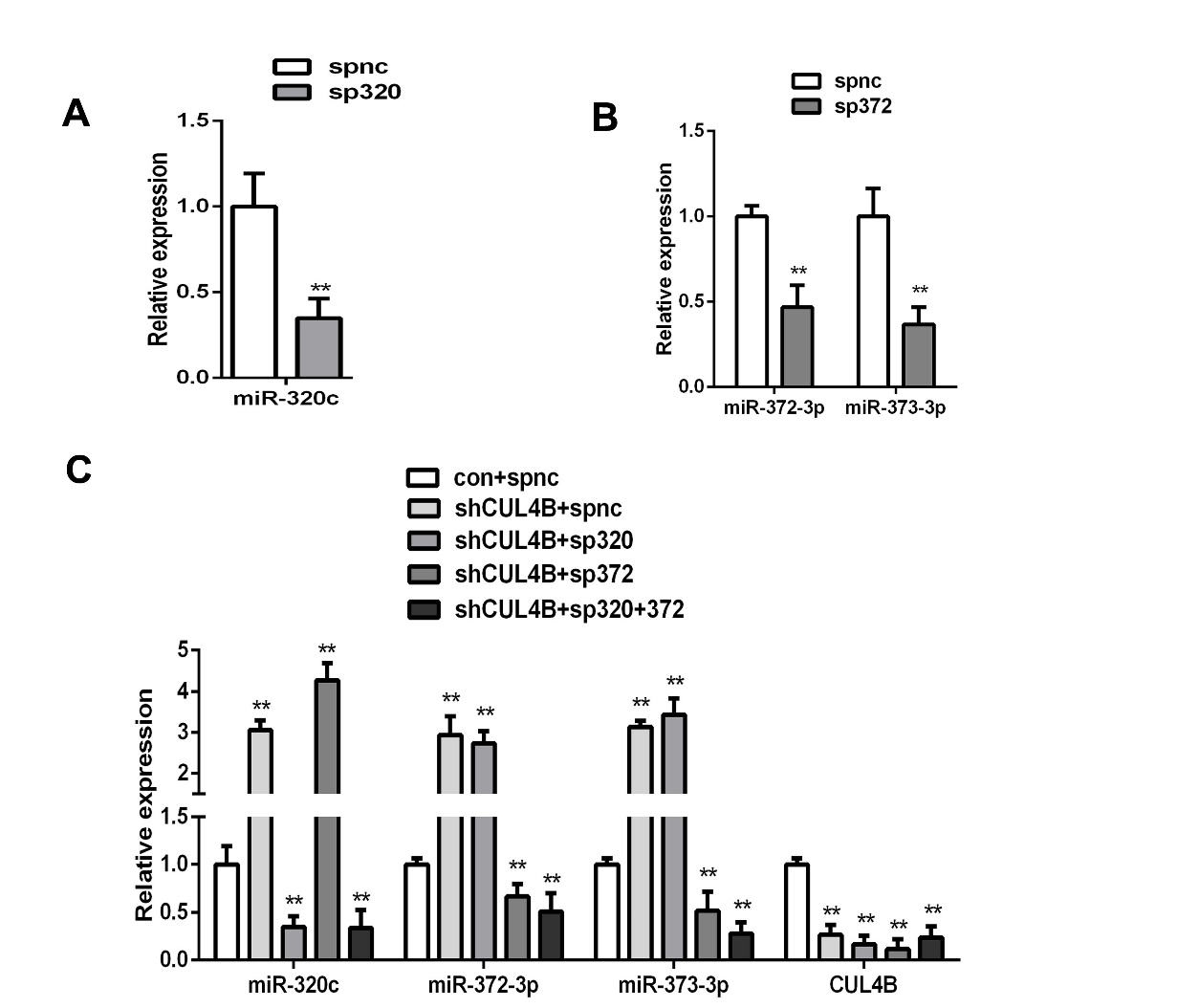
**

**Fig. S4** (**A**, **B)** Stable expression miR-320c sponge (sp320c), miR-372-3p/373-3p sponge (sp372) and corresponding control sponge (spnc) in PDLSCs using lentiviral expression vector. The expression ofmiR-320c or miR-372/373-3p in indicated PDLSCs were determined by qRT-PCR. **(C)** Stable expression of miR-320c sponge (sp320c), miR-372-3p/373-3p sponge (sp372) and corresponding control sponge (spnc) in CLU4B stable knockdown (shCUL4B) together with control PDLSCs (con) PDLSCs using lentiviral expression vector, indicated cells were cultured in osteogenic medium for 21 days, the expression of CUL4B, miR-320c and miR-372/373-3p were analyzed by qRT-PCR. All quantification analyses were based on independent triplicate experiments. Error bars represent SD. Statistical comparisons were made using two-tailed unpaired t-test, *P < 0.05, **P < 0.01 compared with negative control.

**Supplementary Table 1: Direct targeting of RUNX2 by miRNAs**

| miRNAs | Model | Functional characteristics | References |
| --- | --- | --- | --- |
| miR-30d-5p | postmenopausal osteoporosis  colon cancer cell | osteogenic differentiation  tumorigenesis | 1  2 |
| miR-34c | osteosarcoma | tumorigenesis | 3 |
| miR-133a | fracture nonunion | osteoblastogenesis | 4 |
| miR-135b | osteoporosis  Breast Cancer | osteogenic differentiation  tumorigenesis | 5  6 |
| miR-203 | heterotopic ossification  femoral neck fracture breast cancer osteosarcoma | osteogenic differentiation  osteogenic differentiation  tumorigenesis  tumorigenesis | 7 8  6  9 |
| miR-217 | bladder cancer  glioma | tumorigenesis  tumorigenesis | 10  11 |
| miR-320c | mesenchymal stem cells  mesenchymal stem cells | osteogenic differentiation  osteogenic differentiation | 12  13 |
| miR-342 | multiple myeloma | tumorigenesis | 14 |
| miR-363 | multiple myeloma | tumorigenesis | 14 |
| miR-375 | marrow mesenchymal cells | osteogenic differentiation | 15 |
| miR-6797-5p | bone marrow stromal cells | osteogenic differentiation | 16 |

**Supplementary Table 2: Direct targeting miRNAs of CUL4B**

| miRNAs | Model | Functional characteristics | References |
| --- | --- | --- | --- |
| miR-194 | non-small-cell lung carcinoma | tumorigenesis | 17 |
| miR-215 | non-small-cell lung carcinoma | tumorigenesis | 17 |
| miR-192 | non-small-cell lung carcinoma | tumorigenesis | 17 |
| miR-371-373 cluster | cervical cancer  bladder cancer  ovarian cancer | proliferation  tumorigenesis  tumorigenesis | 18  19  20 |
| miR‐34a | colorectal cancer | cancer stemness | 21 |
| miR-125a | gastric cancer | tumorigenesis | 22 |
| miR-33b | gastric cancer | tumorigenesis | 23 |
| miR-200b/c | prostate cancer | cancer stem‐like traits | 24 |
| miR-204 | prostate cancer | tumorigenesis | 25 |

**Supplementary Table 3：Primary antibodies for Western blot**

| **Name** | **Manufacturer** | **Catalog number** |
| --- | --- | --- |
| rabbit anti-CUL4B | Sigma-Aldrich | C9995 |
| Direct-BlotTMHRP anti-GAPDH | BioLegend | 649203 |
| rabbit anti-RUNX2 | abcam | ab236639 |
| rabbit anti-ALP | HUABIO | ET1601-21 |
| rabbit anti-OPN | HUABIO | 0806-6 |

**Supplementary Table 4：Primer sequences used for RT-PCR**

| **Primer name** | **Primer sequence (5' to 3')** |
| --- | --- |
| CUL4B-F | TGCTGCTCAGGAGGTCAGATC |
| CUL4B-R | TGGAATCAAAGTCTTCTCTCTCGTT |
| GAPDH-F | ACAACAGCCTCAAGATCATCAG |
| GAPDH-R | GGTCCACCACTGACACGTTG |
| pri-miR-371-373-F | CCTTCAACAGCTCATCAAGGGCT |
| pri-miR-371-373-R | TACCCGCCCCCTCACCCAATCAA |
| pri-miR-320c1-F | GTTTTGCACTGGTTAATTTTT |
| pri-miR-320c1-R | CATTTTTTTTCTACCCTCTCA |
| pri-miR-320c2-F | GCCTTCTCTTTCCAGTTCTTC |
| pri-miR-320c2-R | GCCACTCTGTATCTCTTCCTA |
| MYC-F | CCCGCTTCTCTGAAAGGCTCTC |
| MYC-R | CTCTGCTGCTGCTGCTGGTAG |
| Nanog-F | CAGAAGGCCTCACACCTAC |
| Nanog-R | ATTGTTCCAGGTCTGGTTGC |
| OCT4-F | CACTGTACTCCTCGGTCCCTTTC |
| OCT4-R | CAGGCACCTCAGTTTGAATGC |
| SOX2-F | CCCAGCAGACTTCACATGT |
| SOX2-R | CCTCCCATTTCCCTCGTTTT |
| RUNX2-F | TCCACACCATTAGGGACCATC |
| RUNX2-R | TGCTAATGCTTCGTGTTTCCA |
| ALP-F | ATGGGATGGGTGTCTCCACA |
| ALP-R | CCACGAAGGGGAACTTGTC |

**Supplementary Table 5：Primer sequences used for ChIP**

| **Primer name** | **Primer sequence (5' to 3')** |
| --- | --- |
| pri-371(-2953~-2609)-F | GCAGTCCTCTTTCAAATGCAC |
| pri-371(-2953~-2609)-R | TCTCGGCTCACCACAACCTTC |
| miR-320c2-F | GTTGAGGAGCACTGGGTATGT |
| miR-320c2-R | CTTACCCTCTCAACCCAGCTT |

**Supplementary Table 6：Primary antibodies for ChIP**

| **Name** | **Manufacturer** | **Catalog number** |
| --- | --- | --- |
| rabbit anti-CUL4B | Sigma-Aldrich | C9995 |
| rabbit anti-EZH2 | BD biosciences | 612667 |
| rabbit anti-H2AK119ub1 | Millipore | #ABE569 |
| normal rabbit IgG | Cell Signaling Technology | #2729 |
| mouse anti-Histone H3 (tri methyl K27) | Abcam | ab6002 |
| mouse anti-DDB1 | Santa Cruz Biotechnology | sc-137132 |
| normal mouse IgG | Santa Cruz Biotechnology | sc-2025 |
| rabbit anti-Histone H3 | Cell Signaling Technology | 4499T |

**Reference**

1. Wu, Z.H., Huang, K.H., Liu, K., Wang, G.T. & Sun, Q. DGCR5 induces osteogenic differentiation by up-regulating Runx2 through miR-30d-5p. *Biochem Biophys Res Commun* **505**, 426-431 (2018).

2. Yu, X., Zhao, J. & He, Y. Long non-coding RNA PVT1 functions as an oncogene in human colon cancer through miR-30d-5p/RUNX2 axis. *J BUON* **23**, 48-54 (2018).

3. van der Deen, M. *et al.* MicroRNA-34c inversely couples the biological functions of the runt-related transcription factor RUNX2 and the tumor suppressor p53 in osteosarcoma. *The Journal of biological chemistry* **288**, 21307-21319 (2013).

4. Peng, H. *et al.* MiR-133a inhibits fracture healing via targeting RUNX2/BMP2. *European review for medical and pharmacological sciences* **22**, 2519-2526 (2018).

5. Chen, B. *et al.* Abnormal expression of miR-135b-5p in bone tissue of patients with osteoporosis and its role and mechanism in osteoporosis progression. *Experimental and therapeutic medicine* **19**, 1042-1050 (2020).

6. Taipaleenmäki, H. *et al.* Targeting of Runx2 by miR-135 and miR-203 Impairs Progression of Breast Cancer and Metastatic Bone Disease. *Cancer research* **75**, 1433-1444 (2015).

7. Tu, B. *et al.* miR-203 inhibits the traumatic heterotopic ossification by targeting Runx2. *Cell death & disease* **7**, e2436 (2016).

8. Yin, Q., Wang, J., Fu, Q., Gu, S. & Rui, Y. CircRUNX2 through has-miR-203 regulates RUNX2 to prevent osteoporosis. *J Cell Mol Med* **22**, 6112-6121 (2018).

9. Huang, Z. *et al.* Knockdown of microRNA-203 reduces cisplatin chemo-sensitivity to osteosarcoma cell lines MG63 and U2OS in vitro by targeting RUNX2. *J Chemother*, 1-14 (2021).

10. Huang, W., Lu, Y., Wang, F., Huang, X. & Yu, Z. Downregulation of circular RNA hsa_circ_0000144 inhibits bladder cancer progression via stimulating miR-217 and suppressing RUNX2 expression. *Gene* **678**, 337-342 (2018).

11. Zhu, Y., Zhao, H., Feng, L. & Xu, S. MicroRNA-217 inhibits cell proliferation and invasion by targeting Runx2 in human glioma. *Am J Transl Res* **8**, 1482-1491 (2016).

12. Hamam, D. *et al.* microRNA-320/RUNX2 axis regulates adipocytic differentiation of human mesenchymal (skeletal) stem cells. *Cell death & disease* **5**, e1499 (2014).

13. Zou, J. *et al.* Resveratrol benefits the lineage commitment of bone marrow mesenchymal stem cells into osteoblasts via miR-320c by targeting Runx2. *Journal of tissue engineering and regenerative medicine* **15**, 347-360 (2021).

14. Gowda, P.S. *et al.* Runx2 Suppression by miR-342 and miR-363 Inhibits Multiple Myeloma Progression. *Mol Cancer Res* **16**, 1138-1148 (2018).

15. Lei, N.B., Liang, X., Wang, P., Liu, Q. & Wang, W.G. Teriparatide alleviates osteoporosis by promoting osteogenic differentiation of hMSCs via miR-375/RUNX2 axis. *European review for medical and pharmacological sciences* **23**, 11043-11050 (2019).

16. Arumugam, B. *et al.* Parathyroid hormone-stimulation of Runx2 during osteoblast differentiation via the regulation of lnc-SUPT3H-1:16 (RUNX2-AS1:32) and miR-6797-5p. *Biochimie* **158**, 43-52 (2019).

17. Mi, J. *et al.* Dysregulation of the miR-194-CUL4B negative feedback loop drives tumorigenesis in non-small-cell lung carcinoma. *Molecular oncology* **11**, 305-319 (2017).

18. Zou, Y. *et al.* CUL4B promotes replication licensing by up-regulating the CDK2-CDC6 cascade

Dysregulation of the miR-194-CUL4B negative feedback loop drives tumorigenesis in non-small-cell lung carcinoma. *The Journal of cell biology* **200**, 743-756 (2013).

19. Liu, X. *et al.* The CUL4B-miR-372/373-PIK3CA-AKT axis regulates metastasis in bladder cancer. *Oncogene* (2020).

20. Duan, P.J., Zhao, J.H. & Xie, L.L. Cul4B promotes the progression of ovarian cancer by upregulating the expression of CDK2 and CyclinD1. *J Ovarian Res* **13**, 76 (2020).

21. Li, Y. *et al.* CUL4B contributes to cancer stemness by repressing tumor suppressor miR34a in colorectal cancer. *Oncogenesis* **9**, 20 (2020).

22. Qi, M. *et al.* CUL4B promotes gastric cancer invasion and metastasis-involvement of upregulation of HER2. *Oncogene* **37**, 1075-1085 (2018).

23. Zhao, M. *et al.* CUL4B/miR-33b/C-MYC axis promotes prostate cancer progression. *The Prostate* **79**, 480-488 (2019).

24. Jiao, M. *et al.* CUL4B regulates cancer stem-like traits of prostate cancer cells by targeting BMI1 via miR200b/c. *The Prostate* **79**, 1294-1303 (2019).

25. Qi, M. *et al.* CUL4B promotes prostate cancer progression by forming positive feedback loop with SOX4. *Oncogenesis* **8**, 23 (2019).
